# The cingulum: anatomy, connectivity and what goes beyond

**DOI:** 10.1093/braincomms/fcaf048

**Published:** 2025-01-31

**Authors:** Linda Kollenburg, Hisse Arnts, Alexander Green, Ido Strauss, Saman Vinke, Erkan Kurt

**Affiliations:** Department of Neurosurgery, Functional Neurosurgery Unit, Radboud University Medical Center, Nijmegen 6525 GA,Netherlands; Department of Neurosurgery, Functional Neurosurgery Unit, Radboud University Medical Center, Nijmegen 6525 GA,Netherlands; Oxford Functional Neurosurgery and Experimental Neurology Group, Nuffield Department of Clinical Neuroscience and Surgery, University of Oxford, Oxford OX39DU, UK; Department of Neurosurgery, Functional Neurosurgery Unit, Tel Aviv Medical Center, Tel Aviv 6801298, Israel; Department of Neurosurgery, Functional Neurosurgery Unit, Radboud University Medical Center, Nijmegen 6525 GA,Netherlands; Department of Neurosurgery, Functional Neurosurgery Unit, Radboud University Medical Center, Nijmegen 6525 GA,Netherlands; Department of Pain & Palliative Care, Radboud University Medical Center, Nijmegen 6525 GA,Netherlands

**Keywords:** cingulum, anatomy, connectivity, cingulotomy, deep brain stimulation

## Abstract

For over half a century, the cingulum has been the subject of neuroanatomical and therapeutic investigations owing to its wide range of functions and involvement in various neurological and psychiatric diseases. Recent clinical studies investigating neurosurgical techniques targeting the cingulum, like deep brain stimulation of the anterior cingulate cortex and cingulotomy, have further boosted interests in this central ‘hub’ as a target for chronic intractable pain. Proper targeting within the cingulum is essential to achieve sufficient pain relief. Despite the cingulum being the centre of research for over a century, its structural and functional organization remains a subject to debate, consequently complicating neurosurgical targeting of this area. This study aims to review anatomical and connectivity data of the cingulum from a clinical perspective in order to improve understanding of its role in pain. For the current study, a systematic literature search was performed to assess the anatomy and functional and structural connectivity of the cingulate bundle and cortex. These outcomes focus on MRI and PET data. Articles were searched within the PubMed database, and additional articles were found manually through reviews or references cited within the articles. After exclusion, 70 articles remained included in this analysis, with 50, 29 and 10 studies describing human, monkey and rat subjects, respectively. Outcomes of this analysis show the presence of various anatomical models, each describing other subdivisions within the cingulum. Moreover, connectivity data suggest that the cingulate bundle consists of three distinct fibre projections, including the thalamocortical, cingulate gyrus and anterior frontal and posterior parietal projections. Further, the cingulum is responsible for a variety of functions involved in chronic pain, like sensory processing, memory, spatial functioning, reward, cognition, emotion, visceromotor and endocrine control. Based on the current outcomes, it can be concluded that the cingulum is a central ‘hub’ for pain processing, because it is a melting pot for memory, cognition and affect that are involved in the complex phenomenon of pain experience, memory, spatial functioning, reward, cognition, emotion, visceromotor and endocrine control. Variability in anatomical and connectivity models complicate proper and standardized neurosurgical targeting, consequently leading to clinicians often being reluctant on stimulation and/or lesioning of the cingulum. Hence, future research should be dedicated to the standardization of these models, to allow for optimal targeting and management of patients with chronic intractable pain.

## Teaser

Kollenburg *et al.* report that the cingulum is a central area connecting various structures of the pain matrix and limbic system, hence raising interests in its potential as a neurosurgical target in patients with chronic intractable pain. Nevertheless, variability in anatomical and connectivity models complicate targeting of the cingulum in clinics.

## Graphical Abstract

**graphical abstract:**
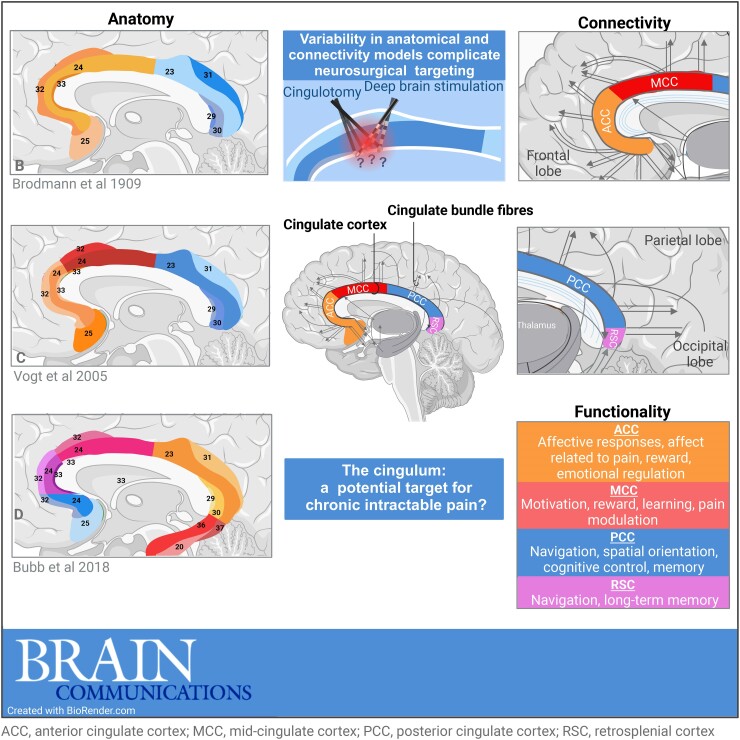

## Introduction

Since the publication of the landmark paper of James Papez in 1937 on the human limbic system and the importance of the cingulate cortex (CC) in the regulation of emotions, the cingulum became a fascinating target to treat a variety of neurological and psychiatric diseases.^1^ The combination of historical outcomes of destructive lesions of the prefrontal lobes with more recent outcomes of stereotactical cingulotomies,^2-4^ neurostimulation studies on deep brain stimulation (DBS)^5,6^ and fundamental animal evidence, have reboosted further interest in this central ‘hub’ as a potential area to treat intractable chronic pain.^7-10^ DBS and cingulotomy are neurosurgical procedures that interfere with the fibre network within the cingulum, allowing for modulation of its activity. In DBS of the anterior CC (DBS-ACC), electrodes are implanted in the cingulum, leading to electrical perturbation of dysfunctional neural circuits. During cingulotomy, lesions are created in the cingulum using a thermocoagulation electrode, consequently causing permanent destruction of its fibre connections.^11^ Following destruction and/or stimulation of the cingulum, a subset of patients report that they are ‘no longer bothered’ by their pain and/or negative emotions, hence suggesting that its potential in the management of chronic pain may be explained by its involvement in emotional and cognitive processing.^3,12,13^ Emotional and cognitive regulation requires the involvement of a complex network including various cortical and subcortical structures such as the thalamus, hippocampus and amygdala. The cingulum contains a distinctive white fibre tract, connecting various cortical and subcortical sites, also referred to as ‘cingulate bundle’.^7^ Recent advancements in tractography have shown that the cingulate bundle extends longitudinally above the corpus callosum^14^ and supports prefrontal, parietal and temporal lobe interactions.^14^ Most fibres joining the cingulate bundle cross the CC, which is considered a principal driver of the cingulate bundle. Throughout the years, various anatomical and functional models have been described for the cingulate bundle and -cortex. Appreciation of the anatomical and connectivity features of the cingulum is of major importance for cingulotomy and DBS-ACC as proper and standardized targeting within the cingulum is required to achieve sufficient pain relief. Moreover, the importance of the latter is also confirmed by studies showing variable outcomes whenever different locations within the cingulum are targeted.^11^

Despite its clinical relevance, understanding of its overall function and the presence of clear visualizations in text books, defining the anatomical and functional details of cingulate bundle and -cortex appears to be rather complex. There is no consensus on the exact borders and fibre tracts within the cingulum, which adds to the difficulty in defining its anatomical and functional properties. Furthermore, only a limited number of studies assessed the anatomy and/or connectivity of the cingulum with respect to chronic intractable pain in particular. As a consequence, the best location for lesioning within the cingulum of patients with chronic pain remains a matter of ongoing debate, thus constraining the achievement of optimal clinical outcomes and proper understanding of the role of the cingulum in pain. Hence, this study aims to review anatomical and structural/functional connectivity data of the cingulum from a clinical perspective, allowing for a better understanding of its involvement in pain.

## Methods

**Figure 1:**
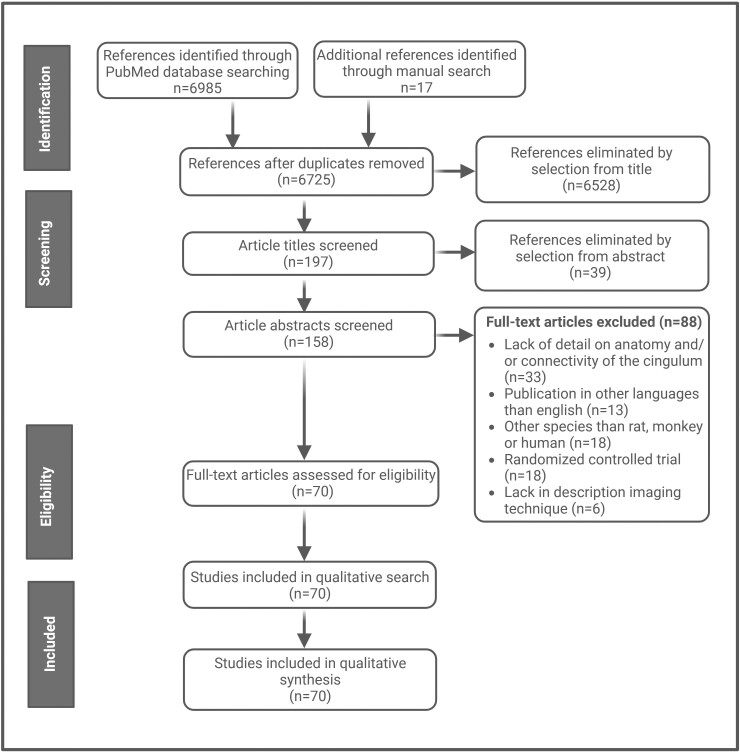
**Literature search.** Overview of literature search performed for the current analysis.

In this systematic literature search, we aim to investigate the role of the cingulum in pain across several outcomes including anatomy, and functional and structural connectivity of the cingulate bundle and -cortex. These outcome measures focus on MRI and PET data. A systematic literature search is performed in PubMed using the following search strategy: [cingulum (tiab) OR cingulate bundle (tiab) OR CC (tiab) OR ACC (tiab) OR MCC (tiab) OR PCC (tiab)] AND [anatomy (tiab) OR anatomy (MeSH) OR functionality (tiab) OR functionality (MeSH) OR connectivity (MeSH) OR connectivity (tiab)] AND [human (tiab) OR human (MeSH) OR monkey (tiab) OR monkey (MeSH) OR rat (tiab) OR rat (MeSH)]. Database filters are applied to refine search results, excluding studies written in languages other than English and randomized controlled trials (Fig. 1). Additional articles were searched manually with Google Scholar, through reviews or references cited within the articles. Reviews that were only used to find additional studies were not included in the current analysis. For studies with extensive data, solely results that correspond with outcomes to our research question are included. Duplicates and/or articles lacking sufficient details on anatomy and/or connectivity of the cingulum are excluded from analysis. After exclusion, 70 articles, published between 1909 and 2023, remained included (Fig. 1), with 50, 29 and 10 studies describing human, monkey and rat subjects, respectively (Supplementary Table 1). These articles can be found under section ‘Anatomy’ and ‘Connectivity’ (Supplementary Table 1). All results compatible with each outcome domain, including anatomy, functionality and structural connectivity, in each study were sought. Studies were searched and/or consulted between the 5 November 2023 and 28 April 2024. All title and abstract records were analysed by two independent reviewers (L.K. and E.K.), and each report retrieved was also analysed by two independent reviewers (L.K. and E.K.). Each study analysed within this literature search is viewed by two independent researchers (L.K. and E.K.) to ensure consensus and the quality of the articles included. In the case of disagreement, additional reviewers (H.A. and S.V.) were consulted. Two reviewers collected data from each report (L.K. and E.K.).

## Results

### Anatomy

For almost half a century, research has been performed on the anatomy of the cingulum. Initially, anatomical data were obtained from tracer studies in the rat/monkey brain. These studies showed heterogeneity in cytoarchitecture, projections and neurotransmitter systems, allowing for the creation of the first anatomical models of the cingulum.^15-18^ However, due to the complex anatomy of the cingulum in humans, it can be expected that these results cannot be directly translated to the human brain. Hence, recent anatomical models, based on newly developed imaging techniques like diffusion-weighted magnetic resonance imaging (dMRI) and functional magnetic resonance imaging, have been described, as these allow for visualization of the cingulum in humans.^7^ DTI is a form of dMRI, which provides detailed *in vivo* visualization and quantification of white matter microstructure. For a pragmatic way of dealing with functional stereotactic operations, it is important to clarify the relevant anatomy and differences between anatomical models of the cingulum and their respective terminologies. For this reason, anatomical data and corresponding models of the cingulum have been summarized in this section.

#### Anatomical models of cingulum

**Figure 2:**
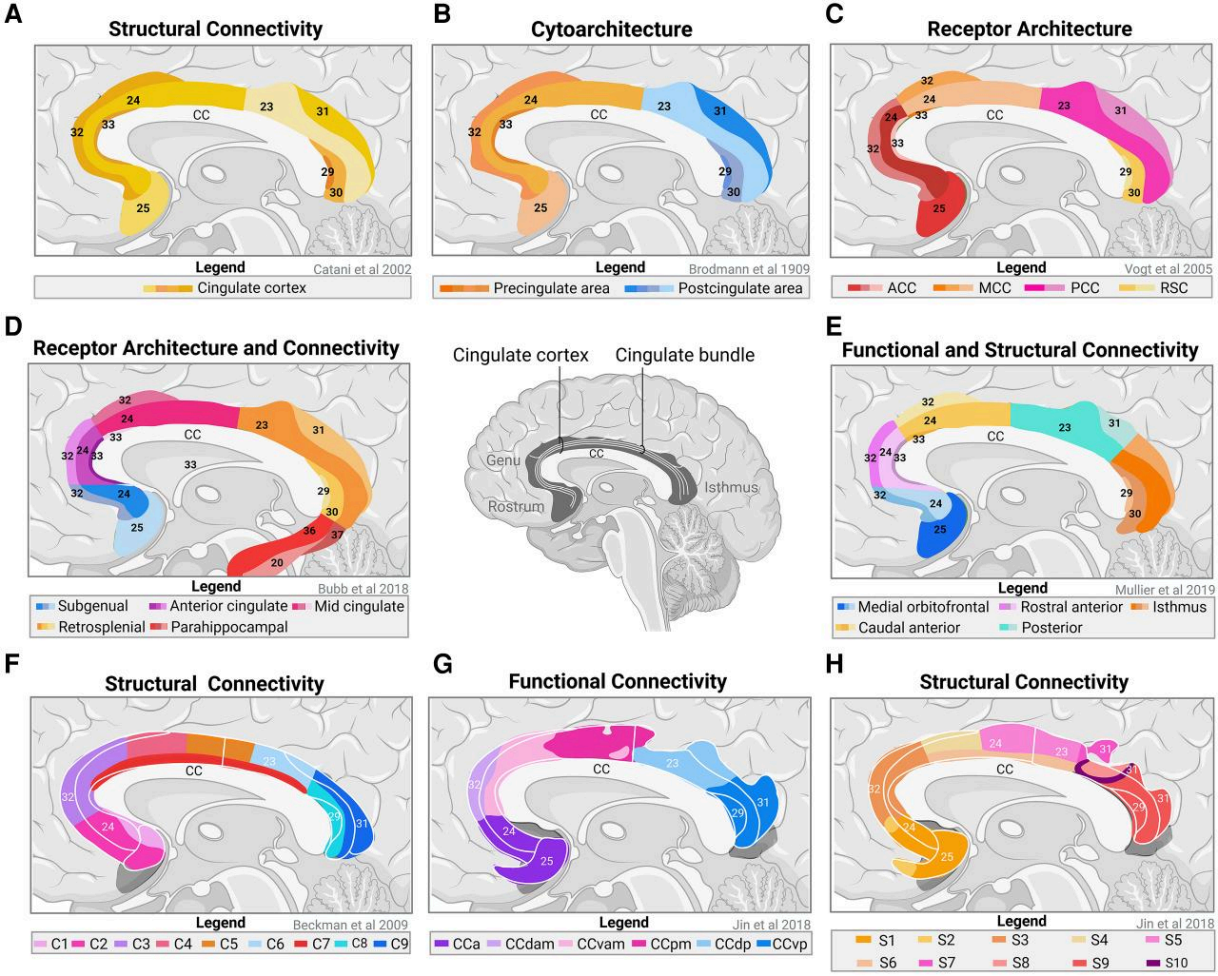
**Anatomical models of cingulate cortex.** Overview of anatomical models of the cingulate cortex. (**A**) Shows the model by Catani *et al.,* which is published in 2002 and used structural connectivity data to suggest that the cingulate cortex is a homogenous structure, without the presence of subdivisions. (**B**) Depicts the model by Brodmann *et al.*, described in 1909, in which cytoarchitecture data are used to divide the cingulate cortex into the precingulate and postcingulate area. (**C**) Illustrates the model by Vogt *et al.*, published in 2005, which used data on receptor architecture to divide the cingulum into 4 subregions consisting of the anterior cingulate cortex (ACC), mid cingulate cortex (MCC), posterior cingulate cortex (PCC) and retro splenial cortex (RSC). (**D**) Shows the model by Bubb *et al.* which is published in 2018 and used data on receptor architecture and connectivity to define a 5-region model of the cingulum, consisting of the subgenual, anterior cingulate, mid cingulate, retrosplenial and parahippocampal cortex. (**E**) Depicts the model by Mullier *et al.*, described in 2019, in which functional and structural connectivity data are used to divide the cingulum into 5-regions including medial orbitofrontal, rostral anterior, caudal anterior, posterior and isthmus. (**F**) Illustrates the model by Beckman *et al.*, published in 2009, which used data on structural connectivity to describe a 9-region model for the cingulate cortex, defined as C1-C9. (**G** and **H**) Show the models by Jin *et al.*, published in 2018, where functional connectivity (**G**) and structural connectivity (**H**) was used to define 6 (CCa-CCvp) (**G**) and 10 (S1-S10) (**H**) region models of the cingulate cortex. The numbers within the models represent the Brodmann areas. Please note that the current drawings show an approximate representation of the models, where slight deviations to the original model might be present. CC, corpus callosum; CCa, anterior cingulate cortex; CCdam, dorsal anterior midcingulate cortex; CCdp, dorsal posterior cingulate cortex; CCpm, posterior midcingulate cortex; CCvam, ventral anterior midcingulate cortex; CCvp, ventral posterior cingulate cortex.

Data obtained from rats and humans both show that the cingulate gyrus consists of the CC and the cingulate fibre bundle that forms the white matter core of the cingulate gyrus.^19^ The CC arches around the corpus callosum on the inner side of the cerebral hemispheres, starting from the rostrum of the corpus callosum, curving around the genu, projecting to the superior portion of the corpus callosum and terminating at the isthmus of the cingulate gyrus (Fig. 2). With regard to the cingulate bundle, it is considered one of the most distinct fibre tracts in the brain.^7,20^ This bundle is located in the deep part of the cingulate gyrus and connects the orbitofrontal cortex (OFC) to the temporal pole.^20^ Initial studies suggested that the cingulum was a unified structure (Fig. 2A).^21^ However, to better describe the heterogeneity in anatomical structure, function and connectivity, the cingulum was later divided into an anterior and posterior region.^22^ Brodmann created the first subdivisions based on the cytoarchitecture. The division consists of a precingulate region, spanning the rostral portion of the cingulate gyrus, and a post-cingulate portion, containing Brodmann areas (BA) 24, 25, 32, 33 and 23, 29, 30, 31, respectively (Fig. 2B).^23,24^ In the following years, von Economo and Koskinas^25^ developed a more detailed model of the cerebral cortex based on cytoarchitecture, describing a total of 107 distinct areas, compared with Brodmann’s 52 areas.^26^ Von Economo and Koskinas used a different system of numbers relative to Brodman and based their model on more detailed cytoarchitecture data including morphology of individual neurons, allowing for identification of specialized neurons and their functions within the CC.^26,27^ Recent insights into these ancient models show that the precingulate subregion was heterogenous in structure and function as distinct patterns of neuronal types, neurotransmitters and fibre connections were found.^15-18,28^ Hence, Vogt *et al.* propose a four-region model based on structural and functional organizations. They subdivide the pre- and post-cingulate areas into the anterior cingulate (ACC), midC (MCC), posterior cingulate cortex (PCC) and retrosplenial cortex (RSC; Fig. 2C).^29^ The ACC contains BA 24, 25, 32 and 33 and can be further divided into the subgenual (sACC/sgACC), perigenual (pACC/pgACC) and dorsal (dACC) region. Although the dACC is described by some to be the same as the MCC, the medial portion has been separately divided into the anterior (aMCC) and posterior (pMCC) areas, covering BA 24, 32 and 33.^30^ The PCC, on the other hand, houses BA 23 and 31 and can be further divided into the ventral (vPCC) and dorsal (dPCC) part, whereas the RSC includes small portions of BA 29 and 30.^18,19^ With this model, it is suggested that the ACC and MCC are most predominantly involved in pain as these areas mediate pain processing in two cognitive domains with sensory-discriminative and affective-motivational components.^29^ In opposite to the model as described by Vogt and colleagues, others use data on fibre orientation density, obtained from dMRI, and define three distinct subdivisions including the parahippocampal, subgenual and retrosplenial area.^14,31^ However, Heilbronner and Haber suggest that, due to the length and complexity of the cingulate bundle fibres, its anatomical subdivisions should be based on the frontal cortical and subcortical projections as more detailed subdivisions might correspond better with functional alterations. Hence, their study proposes a four-region model instead, distinguishing the: rostral subgenual, rostral dorsal/anterior cingulate, caudal dorsal/retrosplenial and temporal/parahippocampal area.^32^ Bubb *et al.* recommends adding the midcingulate cortical area to the 4-region model of Heilbronner and Haber, resulting in 5 subdivisions: subgenual, anterior cingulate, mid cingulate, RSC and parahippocampal, with each region having its own fibre branches (Fig. 2D).^32^ In contrast, Mullier *et al.*^33^ uses data on functional connectivity in patients with early psychosis and describes the presence of five other subdivisions including the medial orbitofrontal, rostral anterior, caudal anterior, posterior and isthmus (Fig. 2E). Other studies use DTI and added additional subdivisions of the CC, defining another four-region model (anterior, middle, posterior and parrahippocampal area),^34^ six-region model (rostral anterior, caudal anterior, posterior cingulate, isthmus cingulate, parahippocampal and entorhinal cortex)^35^ and nine-region model (Cluster 1–9; Fig. 2F).^36^ Moreover, Kennis *et al.*^37^ use dMRI and define alternative subdivisions within the cingulum, which include the rostral, caudal, posterior, isthmus and parahippocampal area. More recently, Jin *et al.*^38^ performed resting state functional MRI and structural DTI, dividing the CC into six regions (Fig. 2G), which can be further divided into 10 subregions, referred to as S1–S10 (Fig. 2H). Another study even defines a greater number of subdivisions with at least 13 regions being distinguished in the CC of rats. Their study suggests further division of the ACC and RSC into dorsal and ventral areas.^39^

### Connectivity and function

Not only data on anatomy but also outcomes covering connectivity and functionality features of the cingulum contribute to the ongoing discussion on the most optimal location for targeting within the cingulum in patients with chronic pain. In order to further explore the clinical potential of this central ‘hub’ for patients with intractable pain, all these data should be integrated with one another. Hence, functional and connectivity data have been summarized and visualized for various anatomical subdivision of the CC.

#### Cingulate cortex

The CC is considered a principal driver of the cingulum due to its abundant connections with various cortical and subcortical areas. Owing to the latter, the cingulum is thought to play an important role in pain processing as this is determined by a complex interplay between various functions like attention, emotion, behaviour, memory, reward and learning. To illustrate, pain perception strongly depends on the emotional state and can lead to the formation of long-lasting memories, which in turn contribute to alterations in pain behaviour and overall cognitive functioning.^40^ Fibres originating from the CC and reaching the OFC, insula and basal ganglia are implicated in emotional processing, whereas the pathways connecting the hippocampus and amygdala are thought to be involved in long-term memory as well as emotional processing.^19^ Furthermore, additional fibres terminating in the parietal lobes and lateral prefrontal (lPFC) cortex are thought to be responsible for orienting attention, and executive control, memory and learning, respectively. Moreover, the network formed between the CC, and primary and/or supplementary motor cortices, eye fields and spinal cord is thought to be involved in motor control.^41^ With regard to targeting the cingulum for chronic pain, it is suggested that CC areas that contain strong connectivity to the precuneus are related to unsuccessful outcomes of cingulotomy and DBS-ACC, whereas connections with the thalamus, insula and brainstem through the medial forebrain are related to more successful outcomes in patients with chronic intractable pain.^11^

##### ACC

**Figure 3:**
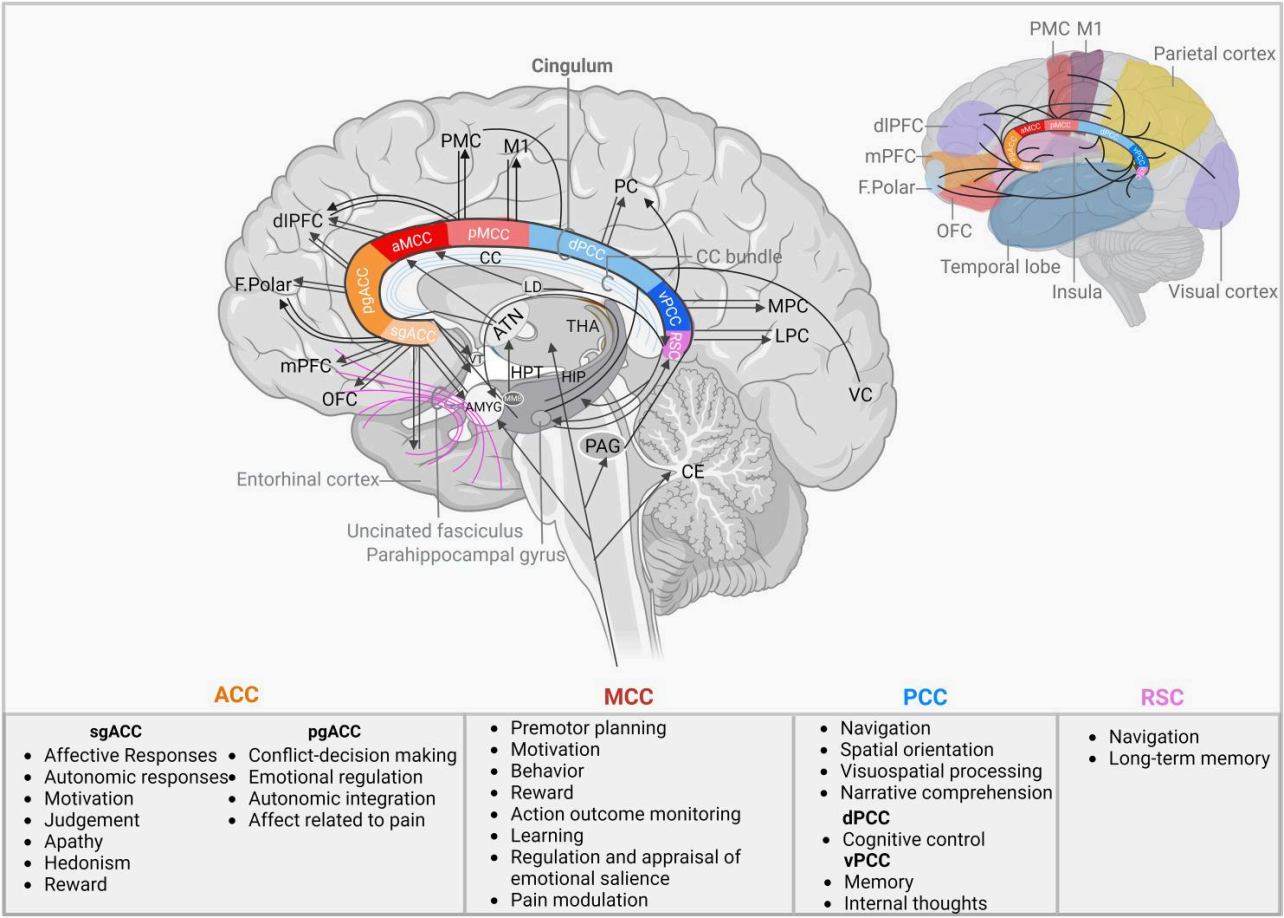
**Connectivity of cingulum.** Overview of the cingulum as a central ‘hub’ with afferent and efferent fibres connecting the subcortical and cortical areas, and corresponding function of the various subdivisions. pgACC, posterior ACC; sgACC, subgenual ACC; MCC, medial cingulate cortex; aMCC, anterior MCC; pMCC, posterior MCC; dPCC, dorsal PCC; vPCC, ventral PCC; PMC, premotor cortex; M1, motor cortex; mPFC, medial prefrontal cortex; OFC, orbitofrontal cortex; F.Polar, frontal pole; dlPFC, dorsolateral prefrontal cortex; ACC, anterior cingulate cortex; RSC, retrosplenial cortex; CC, corpus callosum; HIP, hippocampus; HPT, hypothalamus; MPC, medial parietal cortex; AMYG, amygdala; ATN, anterior thalamic nuclei; LD, lateral dorsal nucleus of thalamus; PAG, periaqueductal gray; THA, thalamus; PC, parietal cortex; PCC, posterior cingulate cortex; MMB, mamillary body; VC, visual cortex; LPC, lateral parietal cortex; VT, ventral lateral nucleus of thalamus; CE, cerebellum.

**Figure 4:**
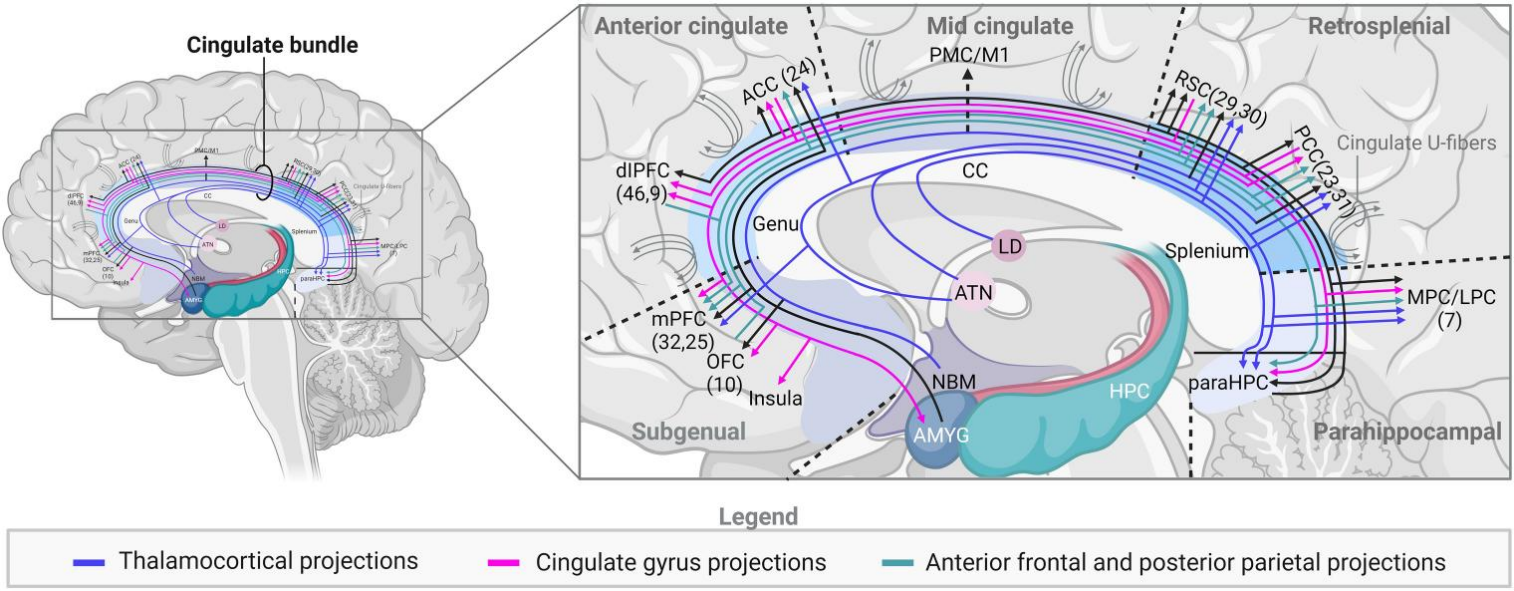
**Fibre projections of cingulate bundle.** Overview of fibre projections of the cingulate bundle, divided into three distinct groups as defined by Mufson and Pandya 1984. The numbers between brackets refer to the corresponding Brodmann areas. PMC, premotor cortex; M1, motor cortex; mPFC, medial prefrontal cortex; OFC, orbitofrontal cortex; dlPFC, dorsolateral prefrontal cortex; ACC, anterior cingulate cortex; RSC, retrosplenial cortex; CC, corpus callosum; paraHPC, parahippocampus; MPC, medial parietal cortex; AMYG, amygdala; ATN, anterior thalamic nuclei; LD, lateral dorsal nucleus of thalamus; NBM, nucleus basalis of Meynert; PCC, posterior cingulate cortex; LPC, lateral parietal cortex.

The ACC is part of the ventromedial prefrontal cortex, consisting of the cingulate sulcus and gyrus dorsal to the corpus callosum and ventral to the superior frontal gyrus.^42^ The ACC is most predominantly connected with the amygdala and is important for emotion and visceromotor and endocrine control,^18,43^ next to memory and reward related functions.^44^ It has been subdivided into subgenual (sACC/sgACC), pregenual (pACC/pgACC) and dorsal ACC (dACC) subregions.^44,45^ The sACC is involved in arousal mechanisms including affective and autonomic responses via projections of the medial prefrontal cortex (mPFC) and OFC to the amygdala, insula, hypothalamus and ventral striatum (Fig. 3).^18,36,44,46,47^ Moreover, other studies show the presence of a structural network consisting of inhibitory and excitatory projections from the hippocampus to the sACC, being most abundant in BA 25.^48,49^ In addition, Choi *et al.*^50^ show the presence of thalamocortical fibres, connecting the anterior nucleus of the thalamus (ATN) with the sACC. Its fibres also cross and reach other bundles such as the uncinated fasciculus and corpus callosum (Fig. 4). These connections, next to connections to medial and anterior thalamus, are associated with motivation, judgement, apathy, hedonism and reward.^7^ Moreover, the sACC also contains fibres from the PCC, dACC, dorsolateral frontal cortex, frontal pole (FP) and entorhinal cortex (Fig. 3).^51^ The sACC (BA 24a) and pACC (BA 24b) are both connected to the thalamus, brainstem, pontine nuclei, tegmental nuclei, superior colliculus, periaqueductal gray (PAG), raphe nuclei, locus coeruleus and reticular formation.^17,48,52^ Fibres from BA 24a and 24b also reach various cortical areas including the retrosplenial, parietal associative and lateral orbitofrontal cortex.^36,52^ The pACC is more functionally connected with areas implicated in conflict decision-making and affective processing, like the entorhinal cortex, sACC, amygdala, PAG, insula, hypothalamus, prefrontal cortex and PCC. These connections allow the pACC to be involved in various processes like emotional regulation, autonomic integration and affect related to pain (Fig. 4).^44,53-56^ Because of its extensive interactions with limbic structures, as well as other areas important for cognition and affective processing, the ACC is thought to form a key component of the pain network.^57,58^ This is supported by evidence showing hyperactivation of the ACC during functional magnetic resonance imaging studies of individuals with neuropathic pain.^40^ Moreover, activation of the ACC is also related to enhanced sadness, which likely suggests that the ACC does not only affect pain via direct nociception and processing of pain signals, but also by inducing the unpleasantness of chronic pain.

##### MCC

Various studies suggest that the MCC is implied in pain modulation and premotor planning with motivational characteristics.^41,59,60^ In the MCC, fibres from all frontal, subcortical and cingulate sites are present that use the cingulate bundle to project to the MCC, anterior insula, basal ganglia, thalamic nuclei e.g. ATN and LD (BA 24c), amygdala, sACC, pACC, PCC and medial temporal and—parietal cortex (MPC and MTC), spinal cord, premotor (PMC), M1, SMA, dmPFC, vlPFC and dIPFC (Fig. 3).^7,17,18,32,44,60-63^ The network containing fibres to motor-related areas is also referred to as cingulate motor area. The connections with the amygdala, sACC and pACC are thought to be important in nociception as these fibres relay information from the reward system. Fibres reaching the SMA mediate action-outcome monitoring and learning (Fig. 3).^60,64,65^ Lastly, the network between the MCC and anterior insula is involved in the regulation and appraisal of emotional salience (Fig. 3).^60,66^ The MCC can be subdivided into anterior (aMCC) and posterior (pMCC), with each portion having its own connections and functions. The aMCC, also referred to as dACC, is active during fear and is therefore also likely to be involved in the formation of long-lasting fear memories arising from acute pain. The aMCC has a role in reward coding of behaviour and the ideomotor mechanism owing to its connections with the amygdala, dorsal striatum, dlPFC and receiving projections from the thalamic nuclei.^36,44,47,63^ The pMCC is more driven by passive movements and contains fibres reaching the dorsal striatum, precentral cortex, dlPFC, parietal and PMC.^18,36,47^

##### PCC

The PCC is has a variety of functions like navigation, spatial orientation, visuospatial processing and narrative comprehension, however, is less involved in pain processing.^67,68^ Its fibres receive cortical input from the thalamic nuclei e.g. anterior, ventral and intralaminar nuclei, and caudal portion of the temporoparietal junction and superior temporal gyrus.^47,48,69-71^ The PCC can be further divided into the dorsal (dPCC) and ventral (vPCC). The vPCC is involved in memory and internal thoughts, due to its fibres connecting the hippocampus, medial temporal lobe and ventromedial PFC e.g. sACC (Fig. 3).^17,72-74^ The dPCC, on the other hand, engages in cognitive control and is activated while performing tasks. Unlike the vPCC, the dorsal portion contains less strong connections to the limbic and paralimbic regions and is therefore assumed to have only minimal interaction with the pain matrix.^67^ The dPCC receives cortical afferents from the parietal-, prefrontal-, premotor- and auditory cortex, inslula, parahippocampal gyrus, visual areas and ACC (Fig. 3).^36,74-76^ Both portions of the PCC have strong connections with the ACC (24a and b), frontal- e.g. dlPFC (BA 46) and FP (BA 10–11), temporal- and parietal lobe.^73^

##### RSC

The RSC is mostly active during the resting state and has a role in spatial functioning including navigation and long-term memory.^18,29,77^ The RSC has strong afferents from the thalamic nuclei like AV, AD and LD and projects towards the brainstem and PAG.^78^ Imaging studies also found a network between the RSC, medial temporal lobe and ventromedial prefrontal cortex e.g. sACC (Fig. 3).^17,72,73^ The fibre bundles in the RSC, are similar to the PCC although it contains only few connections with the amygdala, dlPFC and OFC (Fig. 4).^32^ Interestingly, subjects suffering chronic pain have displayed increased functional connectivity between the RSC and PFC.^79^ Despite an absence of anatomical subdivisions for the RSC, variability in connectivity allows for its subdivision into BA 29 and BA 30. BA 29 of the RSC contains stronger afferents from the motor cortex, visual cortex, BA 24, 25, 11, 48, 49, and the subiculum. Furthermore, BA 30 in the RSC connects with BA 23, PFC, entorhinal cortex, thalamus, posterior PHG presubiculum and temporo-parieto-occipital junction.^80^

#### Cingulate bundle

Throughout the years, various techniques, ranging from microdissections and reconstructions of cellular and white matter stains to non-invasive dMRI have been used to define the fibre tracts of the cingulum. The cingulate bundle contains short and long fibres radiating across the tract, reaching cortical and subcortical sites. The short cortical fibres are also referred to as ‘U-fibres’ and form a link between the medial parts of the frontal, parietal and temporal lobes (Fig. 4).^81^ Moreover, the cortico-cortical and subcortical connections appear to be mostly connected to the cingulate gyrus and join the long axis. The projections to subcortical sites more typically cross the bundle (Fig. 4).^7^ The cingulate bundle has projections to the hippocampus, insula, amygdala and cortical areas such as the mPFC, frontopolar cortex (F.Polar), dIPFC, MPC and lateral parietal cortex (LPC) (Fig. 4).^7^ Based on the afferents and efferents of these fibres, Mufson and Pandya^82^ identified three major groups of connections in the cingulate bundle. The first group consists of the thalamocortical projections. These fibres arise from the LD and ATN, and terminate in the ACC (BA 24), mPFC (BA 32, 25), RSC (BA 29,30), and PCC (BA 23). The second group includes the cingulate gyrus projections, arising from the PCC (BA 23) and ACC (BA 24), terminating in the parrahippocampal-, parietal- (MPC/LPC) (7) and prefrontal cortex, as well as insula, amygdala and PCC (Fig. 4). The last group comprises the projections from the anterior frontal and posterior parietal regions with fibres originating from the dlPFC (BA 46, 9) and OFC (BA 10), moving towards the mPFC, PCC (BA 23, 31), RSC (29, 30) and parietal cortex (MPC/LPC; Fig. 4).^7^

## Discussion

Pain is a complex phenomenon, which is determined by the interaction between various cognitive, social and emotional functions. The current analysis shows that fibres of this complex pain matrix converge in the cingulum (Fig. 3). For this reason, the cingulum is considered a promising target for clinical interventions like cingulotomy and DBS-ACC. Appreciation of the anatomical and functional characteristics of the cingulum is of major importance to optimize clinical outcomes of cingulotomy and DBS-ACC. However, its structural and functional organization remain a subject of debate and should therefore be further investigated.

The cingulate anatomy is of major importance when defining the best target for lesioning and/or destruction. The current analysis shows the presence of various anatomical models for the cingulum, with each model describing different subregions. Not only between, but also within models discrepancies are present. To illustrate, articles covering the four-region model as proposed by Vogt *et al.*, include different BA’s into their description of various subregions. With regard to the PCC, Palomero-Gallagher *et al.*^18,^ suggests that this region consists of BA 23 and 31, whereas Jumah *et al.*^19^ mentions BA 29 and 30 for this area as well. The latter can likely be attributed to the inclusion of additional prelimbic and infralimbic areas into the subdivision of the ACC and PCC in models based on anatomy on connectivity and cytoarchitectonic data.^28,33,37,83^ As a result, large portions of BA 32 and 31 are included in the anatomical models of the cingulum, thus complicating the identification of the borders surrounding the cingulum. The presence of this issue is confirmed in our analysis as recent studies suggest that the posterior portion of the cingulum extends dorsally and rostrally along the callosal sulcus whereas the initial model by Brodmann restricts this portion to the most caudal part of the cingulate gyrus.^18^ Moreover, this study also shows an absence in consensus of the terminologies used to describe the subdivisions. Whereas some authors use ‘aMCC’ to indicate the dorsal portion of the ACC, others use ‘dACC’, ‘acACC’ and/or ‘ adACC’. In addition, studies remain inconclusive on whether the dACC is a part of the ACC or MCC. Whereas the original model, as proposed by Brodmann, does not separate the dACC from the ACC, recent insights suggests that dACC is fundamentally different from the ACC and should therefore be considerd part of the MCC.^18^ Taken together, due to an absence in consensus on the exact borders within and surrounding the cingulum, the anatomical structure of the cingulum could be viewed as a gradient rather than well-defined areas. Consequently, surgical targeting of the cingulum has become more challenging.

Another important aspect for target localization in chronic pain, is connectivity of the cingulum. For the last few decades, studies have used tractography to obtain connectivity data of the cingulum.^21,84^ Despite tractography techniques being improved over the last few years, its accuracy remains questionable for distinct subdivisions of the cingulum, as these might appear united due to the presence of short-fibres providing overlap in the trajectories.^7^ With regard to tractography, Beckmann *et al.*^36^ describes usage of a threshold for their analysis on cingulate connectivity outcomes, causing voxels present in <2 subjects to be excluded. Although this measure may improve the accuracy of the results, it might also lead to an underestimation of the cingulate bundle connections. Hence, data obtained from tractography requires careful interpretation. Moreover, many functional imaging studies have focused on transparent mapping between the motor output and sensory input. Consequently, functional mapping of regions such as the PCC has become more challenging due to an absence of direct connections between the primary sensory and cortical regions.^67^ Consequently, these limitations in imaging likely contribute to the presence of discrepancies in connectivity and functionality data of the cingulum. Whereas Bentley *et al.* suggest the engagement of the PCC in pain processing, others strictly limit its function to low-level sensory or motor processing, due to an absence of connections to the primary sensory and motor areas.^36,67^ Furthermore, various studies describe the presence of additional fibres from the vPCC towards the medial temporal lobe and ventromedial PFC e.g. sACC,^17,72-74^ while others only suggest connectivity with the hippocampus.^36^ Despite the development of imaging techniques, allowing for connectivity analysis in vivo in humans, most studies perform their analysis on non-human primates or rodents, which likely contribute to the inconsistencies in functionality outcomes.^74^ All in all, despite advancements in imaging techniques improving the localization of the cingulate bundle fibres, its exact projections remain estimations.

Despite challenges in imaging of the cingulate bundle, current outcomes raise interests in the cingulum as a neurosurgical target, owing to its extensive fibre connections with structures of the pain matrix and limbic system like thalamus and sensory cortex (Fig. 3). Added to that, clinical outcomes from our review on cingulotomy and DBS-ACC, showing substantial pain relief in >43% and >57% of patients with chronic intractable pain, respectively,^11,57^ also highlight involvement of the cingulum in chronic pain. Despite these promising outcomes, discrepancies in anatomical and connectivity data complicate use of the cingulum as a neurosurgical target, as physicians select different locations of the cingulum for lesioning and/or stimulation. Whereas both techniques mention targeting of the ACC for chronic intractable pain, it seems that for cingulotomy, the anterior mid-CC (aMCC) and for DBS-ACC, the cingulate bundle rather than the CC is targeted.^11^ Despite this lack of consensus for targeting, functionality study outcomes suggest that targeting at the PCC^68,85^ and/or dACC^45,86^ might have the highest clinical potential in the treatment of chronic intractable pain as these regions were most activated upon exposure of thermal pain stimuli. With regard to activation of the PCC, most studies do not provide an explanation for this finding; however, Gelnar *et al.*^87^ attribute the occurrence to the presence of direct nociceptive spinothalamic inputs to the mid-cingulate motor region and the caudal visuospatial regions. As limited studies describe the involvement of the PCC in chronic pain, with these spinothalamic connections only being found in monkeys and cats so far, conclusions requires careful interpretations. In opposition to the PCC, plenty of evidence has supported the involvement of the dACC in pain processing during subacute and chronic pain states.^45,60,66,86^ Based on our results and clinical experience, we propose targeting the dACC in cases of chronic intractable pain, due to its strong connections with cortical and subcortical areas involved in nociceptive processing (e.g. anterior insula, basal nuclei, thalamic nuclei, amygdala and dlLPFC; Fig. 4).^11,57^ When compared with other subregions, the dACC has the highest bundle density as the majority of fibres from all frontal, subcortical and cingulate sites, joining the cingulate bundle, and cross this area. Added to that, the ACC contains Von Economo neurons (VENs), which are oval or spindle-shaped neurons with highly specialized functions. Due to the specific distribution of these neurons, which is limited to the ACC and fronto-insular cortex, it is thought to functionally be connected to the limbic system and may therefore contribute to regulating behaviour, emotion and pain within the ACC.^27^ Outcomes by Kwan *et al.*^58^ describing that activation of the ACC in more patients compared to the PCC upon pain-related activation (80% and 50%, respectively) also support neurosurgical targeting of more anterior portions of the cingulum in patients with chronic intractable pain. Although Bromm *et al.*^70^ support the involvement of the ACC in pain processing, they mention that functionality outcomes of PET and functional magnetic resonance imaging might have been too low visualize activation of the PCC, which might be due to the lower fibre density. With regard to the models of the anatomical divisions of the cingulum, we suggest that the four-division model as proposed by Brodmann, is clinically most useful as visualization of additional subdivisions will be challenging during surgery. Altogether, consensus on the anatomy and connectivity of the cingulum is required to optimize clinical outcomes of DBS-ACC and cingulotomy for patients with chronic intractable pain. Hence, future research should be dedicated to the standardization of these models.

Though the current analysis was performed to the best of our expertise, several limitations are present. Regarding anatomical and connectivity models of the cingulum, various issues should be taken into consideration like data being obtained from various animal species, and the presence of pathophysiology in study subjects. As the organization of brain structures and connections differ among species, this might have led to bias in the proposed model and outcomes. However, as restricted data are available for human subjects with animal models allowing for a deeper understanding while sharing overlapping anatomical and connectivity properties, and only minimal animal studies being included, we choose to cover these as well in the current analysis. Moreover, a subset of studies included patients with various aetiologies e.g. schizophrenia,^55^ which possibly interfered with our results as connections might be altered in these pathologies. We were aware of this issue prior to analysis; however, as very few subjects were suffering from a disease during analysis, with most being part of a bigger sample with healthy individuals, providing solely a generalized analysis on the cingulum, inclusion criteria could not be adjusted for this. In addition, variation between individuals due to gender and/or age might also diminish the accuracy of our results as various studies have shown alterations in connectivity in older subjects and differences in the structure of the cingulum in men versus women.^7,47,88^ Another factor complicating our analysis includes terminologies such as ‘cingulate bundle’ and ‘CC’ being used interchangeably, despite both being separate structures within the cingulum. Further, various studies point out the presence of an asymmetrical division of the cingulum.^88^ Important to note is that models within our figures were drawn in an unilateral sagittal manner and that differences due to lateralization may have been present, hence, models likely would have appeared differently if these had been depicted from the opposite hemisphere. Prospective studies should be focusing on defining a standardized anatomical and connectivity model of the cingulum for clinics, in order to facilitate current neurosurgical targeting of the cingulum and optimize efficacy. Owing to the fact that efficacy of cingulotomy and DBS-ACC is in part achieved by altering connectivity to various structures of the pain matrix and limbic system through the cingulum, alongside the extensive involvement of these networks in pain processing, investigating alternative neurosurgical strategies and targets would be valuable. For this matter, neurosurgical procedures like thalamotomy and magnetic resonance guided focused ultrasound (MRgFUS), targeting other areas within these networks, also form an interesting field for future research.

### Anatomy and connectivity in clinical targeting of the cingulum

Outcomes on anatomy and connectivity properties of the cingulum are of major importance when it comes to targeting of the cingulum in patients with chronic intractable pain. Owing to the fact that the cingulum is a large structure with heterogeneity in both connectivity and anatomy throughout, targeting this area poses significant challenges. This underscores the importance of comprehensive anatomical and connectivity data, which can guide more precise and effective neurosurgical targeting of the cingulum of patients with chronic pain. Studies on the gross anatomy of the cingulum focus on structural organizations on the macroscopic level. These form a fundamental base into defining basic subdivisions within the cingulum, leading to the creation of different anatomical models. Anatomical models facilitate standardization and enhance precision in targeting as clinicians use these models to specify the exact subregion that was or should be targeted rather than referring to the cingulum as a whole, making targeting more accurate. To date, the model by Vogt *et al.*, dividing the CC into four areas (ACC, MCC, PCC, RSC), is most frequently used in clinics. The ACC seems to be the most interesting when it comes to pain as this area is most frequently targeted in studies showing significant improvements in chronic pain.^11^ It should be noted that though data on the gross anatomy of the cingulum is sufficient to create basic anatomical models that facilitate guidance in clinical targeting, microanatomical data are required for more accurate identification of targets and subdivisions into more detailed anatomical models of the cingulum. Studies on the microanatomy of the cingulum investigate structural organization of tissues and cells at a microscopic level and therefore enable further refinement of areas initially outlined by the gross anatomy. As a result, this leads to more accurate target descriptions and guidance during neurosurgical targeting of the cingulum. Further, microanatomical data (e.g. receptor distribution and neuronal types) improve understanding of the exact mechanisms of pain within the cingulum and may be of use in the development of future targeted therapies for chronic pain. Microanatomical data enable further distinction of the ACC into sgACC, pgACC and dACC subregions.^44,45^ It seems that the dACC, in particular, is important when it comes to chronic pain, due to the presence of specialized Von Economo neurons and this area being the most activate upon exposure of pain stimuli.^45,86^ Connectivity outcomes provide further insights as to why the dACC is the most interesting in chronic pain, unravelling details about the presence of strong connections of this area with structures involved in nociceptive processing. Data on connectivity suggest that projections in the thalamocortical, cingulate gyrus and anterior frontal and posterior fibre tracts, connecting the dACC with structures of the limbic system and pain matrix (e.g. prefrontal cortex, thalamus, amygdala, hippocampus, PAG) are important when it comes to pain relief. It is hypothesized that neurosurgical techniques like cingulotomy and DBS-ACC can affect cingulum connectivity.^11^ In cingulotomy, lesioning is performed, causing irreversible destruction of fibre connections, whereas in DBS-ACC, electrical stimulation is applied to the cingulate area, causing reversible inhibition of neuronal activity.^11^ Reduced firing through lesioning or stimulation of connections from the dACC with structures of the limbic system and pain matrix is thought to diminish the transfer of painful signals. As a result, this potentially leads to reduced activation of nociceptive systems, followed by improved management of painful sensations.^11^ In clinics, such interference with cingulate connectivity by cingulotomy and DBS-ACC have shown to cause significant decreases in chronic pain scores.^11^ Taken together, gross and micro anatomical data aid in proper targeting through subdividing areas of the CC, whereas connectivity outcomes allow for a different approach in which targets within the cingulate bundle rather than cortex can be identified. Combining anatomical with connectivity data leads to a greater comprehension of the role of certain cingulate areas in pain and thus forms a fundamental aspect in achieving more precise targeting and improved clinical outcomes of neurosurgical techniques like cingulotomy and DBS-ACC.

## Conclusion

There is a growing interest in the cingulum as a neurosurgical target for treating patients with chronic intractable pain. Data on connectivity show that the cingulum is a central ‘hub’ for pain processing, because it is a melting pot for memory, cognition and affect that are involved in the complex phenomenon of pain experience, memory, spatial functioning, reward, cognition, emotion, visceromotor and endocrine control. Despite this recognition, cingulotomy and DBS-ACC do not belong to the standard neurosurgical arsenal for chronic pain, which can likely be attributed to the lack of consensus in anatomical and connectivity models, as this complicates targeting of the cingulum. Hence, future research should be dedicated to the standardization of anatomical and connectivity models for clinics, to allow for optimal targeting and management of patients with chronic intractable pain.

## Supplementary Material

fcaf048_Supplementary_Data

## Data Availability

Data supporting this study are included within the article. Data sharing is not applicable to this article as no new data were created or analysed in this study.
