# Supplementary material for: The cingulum: anatomy, connectivity and what goes beyond
Source: Brain Commun. 2025 Jan 31;7(1):fcaf048. doi: 10.1093/braincomms/fcaf048 (PMC11824423; doi:10.1093/braincomms/fcaf048)
Supplement: fcaf048_Supplementary_Data [file fcaf048_supplementary_data.docx]

| **Number**  **Supplementary Table 1: Overview of included articles** | **Authors (date)** | **Outcome** | **Species** | **Full reference** |
| --- | --- | --- | --- | --- |
| 1 | Braak et al (1976)^1^ | Anatomy | Human | Braak H. A primitive gigantopyramidal field buried in the depth of the cingulate sulcus of the human brain. *Brain research*. 1976;109(2):219-233. |
| 2 | Phan et al (2002)^2^ | Anatomy | Human | Phan KL, Wager T, Taylor SF, Liberzon I. Functional neuroanatomy of emotion: a meta-analysis of emotion activation studies in PET and fMRI. *Neuroimage*. 2002;16(2):331-348. |
| 3 | Vogt et al (1987)^3^ | Anatomy, connectivity | Monkey | Vogt BA, Pandya DN, Rosene DL. Cingulate cortex of the rhesus monkey: I. Cytoarchitecture and thalamic afferents. *Journal of Comparative Neurology*. 1987;262(2):256-270. |
| 4 | Palomero-Gallagher et al (2009)^4^ | Anatomy, functionality, connectivity | Human | Palomero‐Gallagher N, Vogt BA, Schleicher A, Mayberg HS, Zilles K. Receptor architecture of human cingulate cortex: Evaluation of the four‐region neurobiological model. *Human brain mapping*. 2009;30(8):2336-2355. |
| 5 | Bubb et al (2018)^5^ | Anatomy, functionality, connectivity | Human, monkey, rat | Bubb EJ, Metzler-Baddeley C, Aggleton JP. The cingulum bundle: Anatomy, function, and dysfunction. *Neurosci Biobehav Rev*. Sep 2018;92:104-127. doi:10.1016/j.neubiorev.2018.05.008 |
| 6 | Jumah et al (2024)^6^ | Anatomy, functionality, connectivity | Human | Jumah FR, Dossani RH. Neuroanatomy, Cingulate Cortex. *StatPearls*. StatPearls Publishing LLC.; 2024. |
| 7 | Weininger et al (2019)^7^ | Anatomy | Human, monkey, rat | Weininger J, Roman E, Tierney P, et al. Papez’s forgotten tract: 80 years of unreconciled findings concerning the thalamocingulate tract. *Frontiers in neuroanatomy*. 2019;13:14. |
| 8 | Sturm et al (2013)^8^ | Functionality | Human | Sturm VE, Sollberger M, Seeley WW, et al. Role of right pregenual anterior cingulate cortex in self-conscious emotional reactivity. *Soc Cogn Affect Neurosci*. Apr 2013;8(4):468-74. doi:10.1093/scan/nss023 |
| 9 | Catani et al (2002)^9^ | Anatomy | Human | Catani M, Howard RJ, Pajevic S, Jones DK. Virtual in vivo interactive dissection of white matter fasciculi in the human brain. *Neuroimage*. 2002;17(1):77-94. |
| 10 | Budisavljevic et al (2016)^10^ | Anatomy | Human | Budisavljevic S, Kawadler JM, Dell'Acqua F, et al. Heritability of the limbic networks. *Social cognitive and affective neuroscience*. 2016;11(5):746-757. |
| 11 | Brodmann et al (1909)^11^ | Anatomy | Human | Brodmann K. *Vergleichende Lokalisationslehre der Grosshirnrinde in ihren Prinzipien dargestellt auf Grund des Zellenbaues*. Barth; 1909. |
| 12 | Loukas et al (2011)^12^ | Anatomy | Human | Loukas M, Pennell C, Groat C, Tubbs RS, Cohen-Gadol AA. Korbinian Brodmann (1868-1918) and his contributions to mapping the cerebral cortex. *Neurosurgery*. Jan 2011;68(1):6-11; discussion 11. doi:10.1227/NEU.0b013e3181fc5cac |
| 13 | Von Economo et al (1925)^13^ | Anatomy | Human | von Economo CF, Koskinas GN. *Die cytoarchitektonik der hirnrinde des erwachsenen menschen*. J. Springer; 1925. |
| 14 | Triarhou et al (2007)^14^ | Anatomy | Human | Triarhou LC. The Economo-Koskinas atlas revisited: cytoarchitectonics and functional context. *Stereotactic and functional neurosurgery*. 2007;85(5):195-203. |
| 15 | Vogt et al (2005)^15^ | Anatomy | Human, monkey | Vogt BA. Pain and emotion interactions in subregions of the cingulate gyrus. *Nat Rev Neurosci*. Jul 2005;6(7):533-44. doi:10.1038/nrn1704 |
| 16 | Vogt et al (2016)^16^ | Anatomy | Human, monkey, rat | Vogt BA. Midcingulate cortex: structure, connections, homologies, functions and diseases. *Journal of chemical neuroanatomy*. 2016;74:28-46. |
| 17 | Jones et al (2013)^17^ | Anatomy | Human, monkey | Jones DK, Christiansen KF, Chapman RJ, Aggleton JP. Distinct subdivisions of the cingulum bundle revealed by diffusion MRI fibre tracking: implications for neuropsychological investigations. *Neuropsychologia*. Jan 2013;51(1):67-78. doi:10.1016/j.neuropsychologia.2012.11.018 |
| 18 | Tournier et al (2004)^18^ | Anatomy | Human | Tournier J-D, Calamante F, Gadian DG, Connelly A. Direct estimation of the fiber orientation density function from diffusion-weighted MRI data using spherical deconvolution. *Neuroimage*. 2004;23(3):1176-1185. |
| 19 | Heilbronner et al (2014)^19^ | Anatomy, connectivity | Human | Heilbronner SR, Haber SN. Frontal cortical and subcortical projections provide a basis for segmenting the cingulum bundle: implications for neuroimaging and psychiatric disorders. *J Neurosci*. Jul 23 2014;34(30):10041-54. doi:10.1523/jneurosci.5459-13.2014 |
| 20 | Mullier et al (2019)^20^ | Anatomy | Human | Mullier E, Roine T, Griffa A, et al. N-acetyl-cysteine supplementation improves functional connectivity within the cingulate cortex in early psychosis: a pilot study. *International Journal of Neuropsychopharmacology*. 2019;22(8):478-487. |
| 21 | Metzler-Baddeley (2012)^21^ | Anatomy | Human | Metzler-Baddeley C, Jones DK, Steventon J, Westacott L, Aggleton JP, O'Sullivan MJ. Cingulum microstructure predicts cognitive control in older age and mild cognitive impairment. *J Neurosci*. Dec 5 2012;32(49):17612-9. doi:10.1523/jneurosci.3299-12.2012 |
| 22 | Whitford et al (2014)^22^ | Anatomy | Human | Whitford TJ, Lee SW, Oh JS, et al. Localized abnormalities in the cingulum bundle in patients with schizophrenia: a diffusion tensor tractography study. *NeuroImage: Clinical*. 2014;5:93-99. |
| 23 | Beckmann et al (2009)^23^ | Anatomy, functionality, connectivity | Human | Beckmann M, Johansen-Berg H, Rushworth MF. Connectivity-based parcellation of human cingulate cortex and its relation to functional specialization. *Journal of Neuroscience*. 2009;29(4):1175-1190. |
| 24 | Kennis et al (2016)^24^ | Anatomy | Human | Kennis M, Van Rooij S, Kahn R, Geuze E, Leemans A. Choosing the polarity of the phase-encoding direction in diffusion MRI: Does it matter for group analysis? *NeuroImage: Clinical*. 2016;11:539-547. |
| 25 | Jin et al (2018)^25^ | Anatomy | Human | Jin F, Zheng P, Liu H, Guo H, Sun Z. Functional and anatomical connectivity‐based parcellation of human cingulate cortex. *Brain and behavior*. 2018;8(8):e01070. |
| 26 | Jones et al (2005)^26^ | Anatomy | Rat | Jones BF, Groenewegen HJ, Witter MP. Intrinsic connections of the cingulate cortex in the rat suggest the existence of multiple functionally segregated networks. *Neuroscience*. 2005;133(1):193-207. doi:10.1016/j.neuroscience.2005.01.063 |
| 27 | Hayden et al (2010)^27^ | Functionality, connectivity | Monkey | Hayden BY, Platt ML. Neurons in anterior cingulate cortex multiplex information about reward and action. *J Neurosci*. Mar 3 2010;30(9):3339-46. doi:10.1523/jneurosci.4874-09.2010 |
| 28 | Gasquoine (2013)^28^ | Functionality, connectivity | Human | Gasquoine PG. Localization of function in anterior cingulate cortex: from psychosurgery to functional neuroimaging. *Neurosci Biobehav Rev*. Mar 2013;37(3):340-8. doi:10.1016/j.neubiorev.2013.01.002 |
| 29 | Stevens et al (2011)^29^ | Anatomy, functionality, connectivity | Human | Stevens FL, Hurley RA, Taber KH. Anterior cingulate cortex: unique role in cognition and emotion. *J Neuropsychiatry Clin Neurosci*. Spring 2011;23(2):121-5. doi:10.1176/jnp.23.2.jnp121 |
| 30 | Kelly et al (2021)^30^ | Functionality, connectivity | Monkey | Kelly EA, Thomas VK, Indraghanty A, Fudge JL. Perigenual and Subgenual Anterior Cingulate Afferents Converge on Common Pyramidal Cells in Amygdala Subregions of the Macaque. *J Neurosci*. Nov 24 2021;41(47):9742-9755. doi:10.1523/jneurosci.1056-21.2021 |
| 31 | Oane et al (2023)^31^ | Functionality, connectivity | Human, monkey | Oane I, Barborica A, Mindruta IR. Cingulate Cortex: Anatomy, Structural and Functional Connectivity. *J Clin Neurophysiol*. Sep 1 2023;40(6):482-490. doi:10.1097/wnp.0000000000000970 |
| 32 | Finch et al (1984)^32^ | Connectivity | Rat | Finch DM, Derian EL, Babb TL. Afferent fibers to rat cingulate cortex. *Experimental neurology*. 1984;83(3):468-485. |
| 33 | Wang et al (2021)^33^ | Connectivity | Monkey | Wang J, John Y, Barbas H. Pathways for contextual memory: the primate hippocampal pathway to anterior cingulate cortex. *Cerebral cortex*. 2021;31(3):1807-1826. |
| 34 | Choi et al (2018)^34^ | Connectivity | Human | Choi S-H, Kim Y-B, Cho Z-H. Newly observed anterior thalamocortical fiber of the thalamus using 7.0 T super-resolution magnetic resonance tractography and its implications for the classical Papez circuit. *Journal of Neuroradiology*. 2018;45(3):206-210. |
| 35 | Calderazzo et al (2021)^35^ | Connectivity | Monkey | Calderazzo SM, Busch SE, Moore TL, Rosene DL, Medalla M. Distribution and overlap of entorhinal, premotor, and amygdalar connections in the monkey anterior cingulate cortex. *Journal of Comparative Neurology*. 2021;529(4):885-904. |
| 36 | Fillinger et al (2018)^36^ | Connectivity | Rat | Fillinger C, Yalcin I, Barrot M, Veinante P. Efferents of anterior cingulate areas 24a and 24b and midcingulate areas 24aʹ and 24bʹ in the mouse. *Brain Structure and Function*. 2018;223:1747-1778. |
| 37 | Dixon et al (2017)^37^ | Functionality | Human, monkey, rat | Dixon ML, Thiruchselvam R, Todd R, Christoff K. Emotion and the prefrontal cortex: An integrative review. *Psychological bulletin*. 2017;143(10):1033. |
| 38 | Salvadore et al (2010)^38^ | Functionality | Human | Salvadore G, Cornwell BR, Sambataro F, et al. Anterior cingulate desynchronization and functional connectivity with the amygdala during a working memory task predict rapid antidepressant response to ketamine. *Neuropsychopharmacology*. Jun 2010;35(7):1415-22. doi:10.1038/npp.2010.24 |
| 39 | Bliss et al (2016)^39^ | Connectivity | Human | Bliss TV, Collingridge GL, Kaang BK, Zhuo M. Synaptic plasticity in the anterior cingulate cortex in acute and chronic pain. *Nat Rev Neurosci*. Aug 2016;17(8):485-96. doi:10.1038/nrn.2016.68 |
| 40 | Kröger et al (2016)^40^ | Functionality | Human | Kröger IL, Menz MM, May A. Dissociating the neural mechanisms of pain consistency and pain intensity in the trigemino-nociceptive system. *Cephalalgia*. 2016;36(8):790-799. |
| 41 | Gilbertson et al (2021)^41^ | Functionality, connectivity | Human | Gilbertson H, Fang L, Andrzejewski JA, Carlson JM. Dorsal anterior cingulate cortex intrinsic functional connectivity linked to electrocortical measures of error monitoring. *Psychophysiology*. May 2021;58(5):e13794. doi:10.1111/psyp.13794 |
| 42 | Morecraft et al (1992)^42^ | Connectivity | Monkey | Morecraft RJ, van Hoesen GW. Cingulate input to the primary and supplementary motor cortices in the rhesus monkey: evidence for somatotopy in areas 24c and 23c. *Journal of Comparative Neurology*. 1992;322(4):471-489 |
| 43 | Hatanaka et al (2003)^43^ | Connectivity | Monkey | Hatanaka N, Tokuno H, Hamada I, et al. Thalamocortical and intracortical connections of monkey cingulate motor areas. *Journal of Comparative Neurology*. 2003;462(1):121-138. |
| 44 | Michelet et al (2021)^44^ | Connectivity, functionality | Human, monkey | Michelet T, Badets A. The anterior midcingulate cortex might be a neuronal substrate for the ideomotor mechanism. *Experimental Brain Research*. 2021;239(8):2345-2355. |
| 45 | Rolls et al (2019)^45^ | Functionality, connectivity | Human, monkey | Rolls ET. The cingulate cortex and limbic systems for emotion, action, and memory. *Brain Struct Funct*. Dec 2019;224(9):3001-3018. doi:10.1007/s00429-019-01945-2 |
| 46 | Nguyen et al (2014)^46^ | Functionality, connectivity | Human | Nguyen VT, Breakspear M, Cunnington R. Reciprocal interactions of the SMA and cingulate cortex sustain premovement activity for voluntary actions. *Journal of Neuroscience*. 2014;34(49):16397-16407. |
| 47 | Taylor et al (2007)^47^ | Functionality, connectivity | Human | Taylor SF, Stern ER, Gehring WJ. Neural systems for error monitoring: recent findings and theoretical perspectives. *The Neuroscientist*. 2007;13(2):160-172. |
| 48 | Seeley et al (2007)^48^ | Functionality, connectivity | Human, monkey | Seeley WW, Menon V, Schatzberg AF, et al. Dissociable intrinsic connectivity networks for salience processing and executive control. *Journal of Neuroscience*. 2007;27(9):2349-2356. |
| 49 | Leech et al (2019)^49^ | Functionality, connectivity | Human, monkey | Leech R, Smallwood J. The posterior cingulate cortex: Insights from structure and function. *Handbook of clinical neurology*. 2019;166:73-85. |
| 50 | Bentley et al (2002)^50^ | Functionality | Human | Bentley DE, Youell PD, Jones AK. Anatomical localization and intra-subject reproducibility of laser evoked potential source in cingulate cortex, using a realistic head model. *Clin Neurophysiol*. Aug 2002;113(8):1351-6. doi:10.1016/s1388-2457(02)00149-9 |
| 51 | Seltzer et al (2009)^51^ | Connectivity | Monkey | Seltzer B, Pandya DN. Posterior cingulate and retrosplenial cortex connections of the caudal superior temporal region in the rhesus monkey. *Experimental brain research*. 2009;195:325-334. |
| 52 | Aggleton et al (2014)^52^ | Connectivity | Monkey | Aggleton JP, Saunders RC, Wright NF, Vann SD. The origin of projections from the posterior cingulate and retrosplenial cortices to the anterior, medial dorsal and laterodorsal thalamic nuclei of macaque monkeys. *Eur J Neurosci*. Jan 2014;39(1):107-23. doi:10.1111/ejn.12389 |
| 53 | Kobayashi et al (2007)^53^ | Functionality, connectivity | Monkey | Kobayashi Y, Amaral DG. Macaque monkey retrosplenial cortex: III. Cortical efferents. *Journal of Comparative Neurology*. 2007;502(5):810-833. |
| 54 | Parvizi et al (2006)^54^ | Functionality, connectivity | Monkey | Parvizi J, Van Hoesen GW, Buckwalter J, Damasio A. Neural connections of the posteromedial cortex in the macaque. *Proceedings of the National Academy of Sciences*. 2006;103(5):1563-1568. |
| 55 | Leech et al (2014)^55^ | Functionality, connectivity | Human, monkey | Leech R, Sharp DJ. The role of the posterior cingulate cortex in cognition and disease. *Brain*. Jan 2014;137(Pt 1):12-32. doi:10.1093/brain/awt162 |
| 56 | Kobayashi et al (2003)^56^ | Functionality, connectivity | Monkey | Kobayashi Y, Amaral DG. Macaque monkey retrosplenial cortex: II. Cortical afferents. *Journal of Comparative Neurology*. 2003;466(1):48-79. |
| 57 | Oane et al (2020)^57^ | Functionality, connectivity | Human | Oane I, Barborica A, Chetan F, et al. Cingulate cortex function and multi-modal connectivity mapped using intracranial stimulation. *Neuroimage*. 2020;220:117059. |
| 58 | Cona et al (2019)^58^ | Functionality | Human | Cona G, Scarpazza C. Where is the “where” in the brain? A meta‐analysis of neuroimaging studies on spatial cognition. *Human brain mapping*. 2019;40(6):1867-1886. |
| 59 | Vilensky et al (1981)^59^ | Connectivity | Monkey | Vilensky JA, Van Hoesen GW. Corticopontine projections from the cingulate cortex in the rhesus monkey. *Brain research*. 1981;205(2):391-395. |
| 60 | Barrière et al (2019)^60^ | Connectivity | Rat | Barrière DA, Magalhães R, Traoré A, et al. Structural and functional alterations in the retrosplenial cortex following neuropathic pain. *Pain*. 2019;160(10):2241-2254. |
| **61** | **Morris et al (1999)^61^** | **Connectivity** | **Monkey** | **Morris R, Petrides M, Pandya DN. Architecture and connections of retrosplenial area 30 in the rhesus monkey (Macaca mulatta). *Eur J Neurosci*. Jul 1999;11(7):2506-18. doi:10.1046/j.1460-9568.1999.00672.x** |
| **62** | **Yakovlev et al (1961)^62^** | **Connectivity** | **Human** | **Yakovlev PI, Locke S. Corticocortical connections of the anterior cingulate gyrus; te cingulum and subcallosal bundle. *Trans Am Neurol Assoc*. 1961;86:252-6.** |
| **63** | **Mufson and Pandya (1984)^63^** | **Connectivity** | **Monkey** | **Mufson EJ, Pandya DN. Some observations on the course and composition of the cingulum bundle in the rhesus monkey. *J Comp Neurol*. May 1 1984;225(1):31-43. doi:10.1002/cne.902250105** |
| **64** | **Vogt et al (1981)^64^** | **Anatomy** | **Rat** | **Vogt BA, Peters A. Form and distribution of neurons in rat cingulate cortex: areas 32, 24, and 29. *Journal of Comparative Neurology*. 1981;195(4):603-625.** |
| **65** | **Kollenburg et al (2024)^65^** | **Functionality** | **Human** | **Kollenburg L, Kurt E, Arnts H, Vinke S. Cingulotomy: the last man standing in the battle against medically refractory poststroke pain. *PAIN Reports*. 2024;9(2):e1149. doi:10.1097/pr9.0000000000001149** |
| **66** | **Banovac et al (2021)^66^** | **Anatomy** | **Human, monkey,** | **Banovac I, Sedmak D, Judaš M, Petanjek Z. Von Economo neurons–primate-specific or commonplace in the mammalian brain? *Frontiers in Neural Circuits*. 2021;15:714611.** |
| **67** | **Derbyshire et al (2000)^67^** | **Anatomy** | **Human, monkey, rat** | **Derbyshire SW. Exploring the pain “neuromatrix”. *Current review of pain*. 2000;4(6):467-477.** |
| **68** | **Horn et al (2010)^68^** | **Functionality** | **Human** | **Horn DI, Yu C, Steiner J, et al. Glutamatergic and resting-state functional connectivity correlates of severity in major depression - the role of pregenual anterior cingulate cortex and anterior insula. *Front Syst Neurosci*. 2010;4doi:10.3389/fnsys.2010.00033** |
| **69** | **Bromm et al (2004) ^69^** | **Connectivity** | **Human** | **Bromm B. The involvement of the posterior cingulate gyrus in phasic pain processing of humans. *Neuroscience Letters*. 2004/05/06/ 2004;361(1):245-249. doi:**[**https://doi.org/10.1016/j.neulet.2004.01.018**](https://doi.org/10.1016/j.neulet.2004.01.018) |
| **70** | **Kwan et al (2000)^70^** | **Connectivity, functionality** | **Human** | **Kwan CL, Crawley AP, Mikulis DJ, Davis KD. An fMRI study of the anterior cingulate cortex and surrounding medial wall activations evoked by noxious cutaneous heat and cold stimuli. *Pain*. 2000;85(3):359-374** |

**References**

1. Braak H. A primitive gigantopyramidal field buried in the depth of the cingulate sulcus of the human brain. *Brain research*. 1976;109(2):219-233.

2. Phan KL, Wager T, Taylor SF, Liberzon I. Functional neuroanatomy of emotion: a meta-analysis of emotion activation studies in PET and fMRI. *Neuroimage*. 2002;16(2):331-348.

3. Vogt BA, Pandya DN, Rosene DL. Cingulate cortex of the rhesus monkey: I. Cytoarchitecture and thalamic afferents. *Journal of Comparative Neurology*. 1987;262(2):256-270.

4. Palomero‐Gallagher N, Vogt BA, Schleicher A, Mayberg HS, Zilles K. Receptor architecture of human cingulate cortex: Evaluation of the four‐region neurobiological model. *Human brain mapping*. 2009;30(8):2336-2355.

5. Bubb EJ, Metzler-Baddeley C, Aggleton JP. The cingulum bundle: Anatomy, function, and dysfunction. *Neurosci Biobehav Rev*. Sep 2018;92:104-127. doi:10.1016/j.neubiorev.2018.05.008

6. Jumah FR, Dossani RH. Neuroanatomy, Cingulate Cortex. *StatPearls*. StatPearls Publishing; 2024.

7. Weininger J, Roman E, Tierney P, et al. Papez’s forgotten tract: 80 years of unreconciled findings concerning the thalamocingulate tract. *Frontiers in neuroanatomy*. 2019;13:14.

8. Sturm VE, Sollberger M, Seeley WW, et al. Role of right pregenual anterior cingulate cortex in self-conscious emotional reactivity. *Soc Cogn Affect Neurosci*. Apr 2013;8(4):468-474. doi:10.1093/scan/nss023

9. Catani M, Howard RJ, Pajevic S, Jones DK. Virtual in vivo interactive dissection of white matter fasciculi in the human brain. *Neuroimage*. 2002;17(1):77-94.

10. Budisavljevic S, Kawadler JM, Dell'Acqua F, et al. Heritability of the limbic networks. *Social cognitive and affective neuroscience*. 2016;11(5):746-757.

11. Brodmann K. *Vergleichende Lokalisationslehre der Grosshirnrinde in ihren Prinzipien dargestellt auf Grund des Zellenbaues*. Barth; 1909.

12. Loukas M, Pennell C, Groat C, Tubbs RS, Cohen-Gadol AA. Korbinian Brodmann (1868-1918) and his contributions to mapping the cerebral cortex. *Neurosurgery*. Jan 2011;68(1):6-11; discussion 11. doi:10.1227/NEU.0b013e3181fc5cac

13. von Economo CF, Koskinas GN. *Die cytoarchitektonik der hirnrinde des erwachsenen menschen*. J. Springer; 1925.

14. Triarhou LC. The Economo-Koskinas atlas revisited: cytoarchitectonics and functional context. *Stereotactic and functional neurosurgery*. 2007;85(5):195-203.

15. Vogt BA. Pain and emotion interactions in subregions of the cingulate gyrus. *Nat Rev Neurosci*. Jul 2005;6(7):533-544. doi:10.1038/nrn1704

16. Vogt BA. Midcingulate cortex: structure, connections, homologies, functions and diseases. *Journal of chemical neuroanatomy*. 2016;74:28-46.

17. Jones DK, Christiansen KF, Chapman RJ, Aggleton JP. Distinct subdivisions of the cingulum bundle revealed by diffusion MRI fibre tracking: implications for neuropsychological investigations. *Neuropsychologia*. Jan 2013;51(1):67-78. doi:10.1016/j.neuropsychologia.2012.11.018

18. Tournier J-D, Calamante F, Gadian DG, Connelly A. Direct estimation of the fiber orientation density function from diffusion-weighted MRI data using spherical deconvolution. *Neuroimage*. 2004;23(3):1176-1185.

19. Heilbronner SR, Haber SN. Frontal cortical and subcortical projections provide a basis for segmenting the cingulum bundle: implications for neuroimaging and psychiatric disorders. *J Neurosci*. Jul 23 2014;34(30):10041-10054. doi:10.1523/jneurosci.5459-13.2014

20. Mullier E, Roine T, Griffa A, et al. N-acetyl-cysteine supplementation improves functional connectivity within the cingulate cortex in early psychosis: a pilot study. *International Journal of Neuropsychopharmacology*. 2019;22(8):478-487.

21. Metzler-Baddeley C, Jones DK, Steventon J, Westacott L, Aggleton JP, O'Sullivan MJ. Cingulum microstructure predicts cognitive control in older age and mild cognitive impairment. *J Neurosci*. Dec 5 2012;32(49):17612-17619. doi:10.1523/jneurosci.3299-12.2012

22. Whitford TJ, Lee SW, Oh JS, et al. Localized abnormalities in the cingulum bundle in patients with schizophrenia: a diffusion tensor tractography study. *NeuroImage: Clinical*. 2014;5:93-99.

23. Beckmann M, Johansen-Berg H, Rushworth MF. Connectivity-based parcellation of human cingulate cortex and its relation to functional specialization. *Journal of Neuroscience*. 2009;29(4):1175-1190.

24. Kennis M, Van Rooij S, Kahn R, Geuze E, Leemans A. Choosing the polarity of the phase-encoding direction in diffusion MRI: Does it matter for group analysis? *NeuroImage: Clinical*. 2016;11:539-547.

25. Jin F, Zheng P, Liu H, Guo H, Sun Z. Functional and anatomical connectivity‐based parcellation of human cingulate cortex. *Brain and behavior*. 2018;8(8):e01070.

26. Jones BF, Groenewegen HJ, Witter MP. Intrinsic connections of the cingulate cortex in the rat suggest the existence of multiple functionally segregated networks. *Neuroscience*. 2005;133(1):193-207. doi:10.1016/j.neuroscience.2005.01.063

27. Hayden BY, Platt ML. Neurons in anterior cingulate cortex multiplex information about reward and action. *J Neurosci*. Mar 3 2010;30(9):3339-3346. doi:10.1523/jneurosci.4874-09.2010

28. Gasquoine PG. Localization of function in anterior cingulate cortex: from psychosurgery to functional neuroimaging. *Neurosci Biobehav Rev*. Mar 2013;37(3):340-348. doi:10.1016/j.neubiorev.2013.01.002

29. Stevens FL, Hurley RA, Taber KH. Anterior cingulate cortex: unique role in cognition and emotion. *J Neuropsychiatry Clin Neurosci*. Spring 2011;23(2):121-125. doi:10.1176/jnp.23.2.jnp121

30. Kelly EA, Thomas VK, Indraghanty A, Fudge JL. Perigenual and Subgenual Anterior Cingulate Afferents Converge on Common Pyramidal Cells in Amygdala Subregions of the Macaque. *J Neurosci*. Nov 24 2021;41(47):9742-9755. doi:10.1523/jneurosci.1056-21.2021

31. Oane I, Barborica A, Mindruta IR. Cingulate Cortex: Anatomy, Structural and Functional Connectivity. *J Clin Neurophysiol*. Sep 1 2023;40(6):482-490. doi:10.1097/wnp.0000000000000970

32. Finch DM, Derian EL, Babb TL. Afferent fibers to rat cingulate cortex. *Experimental neurology*. 1984;83(3):468-485.

33. Wang J, John Y, Barbas H. Pathways for contextual memory: the primate hippocampal pathway to anterior cingulate cortex. *Cerebral cortex*. 2021;31(3):1807-1826.

34. Choi S-H, Kim Y-B, Cho Z-H. Newly observed anterior thalamocortical fiber of the thalamus using 7.0 T super-resolution magnetic resonance tractography and its implications for the classical Papez circuit. *Journal of Neuroradiology*. 2018;45(3):206-210.

35. Calderazzo SM, Busch SE, Moore TL, Rosene DL, Medalla M. Distribution and overlap of entorhinal, premotor, and amygdalar connections in the monkey anterior cingulate cortex. *Journal of Comparative Neurology*. 2021;529(4):885-904.

36. Fillinger C, Yalcin I, Barrot M, Veinante P. Efferents of anterior cingulate areas 24a and 24b and midcingulate areas 24aʹ and 24bʹ in the mouse. *Brain Structure and Function*. 2018;223:1747-1778.

37. Dixon ML, Thiruchselvam R, Todd R, Christoff K. Emotion and the prefrontal cortex: An integrative review. *Psychological bulletin*. 2017;143(10):1033.

38. Salvadore G, Cornwell BR, Sambataro F, et al. Anterior cingulate desynchronization and functional connectivity with the amygdala during a working memory task predict rapid antidepressant response to ketamine. *Neuropsychopharmacology*. Jun 2010;35(7):1415-1422. doi:10.1038/npp.2010.24

39. Bliss TV, Collingridge GL, Kaang BK, Zhuo M. Synaptic plasticity in the anterior cingulate cortex in acute and chronic pain. *Nat Rev Neurosci*. Aug 2016;17(8):485-496. doi:10.1038/nrn.2016.68

40. Kröger IL, Menz MM, May A. Dissociating the neural mechanisms of pain consistency and pain intensity in the trigemino-nociceptive system. *Cephalalgia*. 2016;36(8):790-799.

41. Gilbertson H, Fang L, Andrzejewski JA, Carlson JM. Dorsal anterior cingulate cortex intrinsic functional connectivity linked to electrocortical measures of error monitoring. *Psychophysiology*. May 2021;58(5):e13794. doi:10.1111/psyp.13794

42. Morecraft RJ, van Hoesen GW. Cingulate input to the primary and supplementary motor cortices in the rhesus monkey: evidence for somatotopy in areas 24c and 23c. *Journal of Comparative Neurology*. 1992;322(4):471-489.

43. Hatanaka N, Tokuno H, Hamada I, et al. Thalamocortical and intracortical connections of monkey cingulate motor areas. *Journal of Comparative Neurology*. 2003;462(1):121-138.

44. Michelet T, Badets A. The anterior midcingulate cortex might be a neuronal substrate for the ideomotor mechanism. *Experimental Brain Research*. 2021;239(8):2345-2355.

45. Rolls ET. The cingulate cortex and limbic systems for emotion, action, and memory. *Brain Struct Funct*. Dec 2019;224(9):3001-3018. doi:10.1007/s00429-019-01945-2

46. Nguyen VT, Breakspear M, Cunnington R. Reciprocal interactions of the SMA and cingulate cortex sustain premovement activity for voluntary actions. *Journal of Neuroscience*. 2014;34(49):16397-16407.

47. Taylor SF, Stern ER, Gehring WJ. Neural systems for error monitoring: recent findings and theoretical perspectives. *The Neuroscientist*. 2007;13(2):160-172.

48. Seeley WW, Menon V, Schatzberg AF, et al. Dissociable intrinsic connectivity networks for salience processing and executive control. *Journal of Neuroscience*. 2007;27(9):2349-2356.

49. Leech R, Smallwood J. The posterior cingulate cortex: Insights from structure and function. *Handbook of clinical neurology*. 2019;166:73-85.

50. Bentley DE, Youell PD, Jones AK. Anatomical localization and intra-subject reproducibility of laser evoked potential source in cingulate cortex, using a realistic head model. *Clin Neurophysiol*. Aug 2002;113(8):1351-1356. doi:10.1016/s1388-2457(02)00149-9

51. Seltzer B, Pandya DN. Posterior cingulate and retrosplenial cortex connections of the caudal superior temporal region in the rhesus monkey. *Experimental brain research*. 2009;195:325-334.

52. Aggleton JP, Saunders RC, Wright NF, Vann SD. The origin of projections from the posterior cingulate and retrosplenial cortices to the anterior, medial dorsal and laterodorsal thalamic nuclei of macaque monkeys. *Eur J Neurosci*. Jan 2014;39(1):107-123. doi:10.1111/ejn.12389

53. Kobayashi Y, Amaral DG. Macaque monkey retrosplenial cortex: III. Cortical efferents. *Journal of Comparative Neurology*. 2007;502(5):810-833.

54. Parvizi J, Van Hoesen GW, Buckwalter J, Damasio A. Neural connections of the posteromedial cortex in the macaque. *Proceedings of the National Academy of Sciences*. 2006;103(5):1563-1568.

55. Leech R, Sharp DJ. The role of the posterior cingulate cortex in cognition and disease. *Brain*. Jan 2014;137(Pt 1):12-32. doi:10.1093/brain/awt162

56. Kobayashi Y, Amaral DG. Macaque monkey retrosplenial cortex: II. Cortical afferents. *Journal of Comparative Neurology*. 2003;466(1):48-79.

57. Oane I, Barborica A, Chetan F, et al. Cingulate cortex function and multi-modal connectivity mapped using intracranial stimulation. *Neuroimage*. 2020;220:117059.

58. Cona G, Scarpazza C. Where is the “where” in the brain? A meta‐analysis of neuroimaging studies on spatial cognition. *Human brain mapping*. 2019;40(6):1867-1886.

59. Vilensky JA, Van Hoesen GW. Corticopontine projections from the cingulate cortex in the rhesus monkey. *Brain research*. 1981;205(2):391-395.

60. Barrière DA, Magalhães R, Traoré A, et al. Structural and functional alterations in the retrosplenial cortex following neuropathic pain. *Pain*. 2019;160(10):2241-2254.

61. Morris R, Petrides M, Pandya DN. Architecture and connections of retrosplenial area 30 in the rhesus monkey (Macaca mulatta). *Eur J Neurosci*. Jul 1999;11(7):2506-2518. doi:10.1046/j.1460-9568.1999.00672.x

62. Yakovlev PI, Locke S. Corticocortical connections of the anterior cingulate gyrus; te cingulum and subcallosal bundle. *Trans Am Neurol Assoc*. 1961;86:252-256.

63. Mufson EJ, Pandya DN. Some observations on the course and composition of the cingulum bundle in the rhesus monkey. *J Comp Neurol*. May 1 1984;225(1):31-43. doi:10.1002/cne.902250105

64. Vogt BA, Peters A. Form and distribution of neurons in rat cingulate cortex: areas 32, 24, and 29. *Journal of Comparative Neurology*. 1981;195(4):603-625.

65. L.Kollenburg EK, H.Arnts, R.Vinke. Cingulotomy: The Last Man Standing in the Battle against Medically Refractory Poststroke Pain. *Pain Reports*. 2024;9(2)

66. Banovac I, Sedmak D, Judaš M, Petanjek Z. Von Economo neurons–primate-specific or commonplace in the mammalian brain? *Frontiers in Neural Circuits*. 2021;15:714611.

67. Derbyshire SW. Exploring the pain “neuromatrix”. *Current review of pain*. 2000;4(6):467-477.

68. Horn DI, Yu C, Steiner J, et al. Glutamatergic and resting-state functional connectivity correlates of severity in major depression - the role of pregenual anterior cingulate cortex and anterior insula. *Front Syst Neurosci*. 2010;4doi:10.3389/fnsys.2010.00033

69. Bromm B. The involvement of the posterior cingulate gyrus in phasic pain processing of humans. *Neuroscience Letters*. 2004/05/06/ 2004;361(1):245-249. doi:<https://doi.org/10.1016/j.neulet.2004.01.018>

70. Kwan CL, Crawley AP, Mikulis DJ, Davis KD. An fMRI study of the anterior cingulate cortex and surrounding medial wall activations evoked by noxious cutaneous heat and cold stimuli. *Pain*. 2000;85(3):359-374.
